# Teaching “medical interview and physical examination” from the very beginning of medical school and using “escape rooms” during the final assessment: achievements and educational impact in Japan

**DOI:** 10.1186/s12909-022-03130-2

**Published:** 2022-01-28

**Authors:** Haruko Akatsu, Yuko Shiima, Harumi Gomi, Ahmed E. Hegab, Gen Kobayashi, Toshiyuki Naka, Mieko Ogino

**Affiliations:** International University of Health and Welfare School of Medicine, Narita, Japan

**Keywords:** Medical interview, History taking, Physical examination, Escape room, Medical students, Medical education, Early exposure, Rapport building, Japan

## Abstract

**Background:**

There is no consensus regarding the best time to teach two fundamental pillars of clinical medicine: medical interview and physical examination. We investigated the impacts of teaching the course “Medical Interview and Physical Examination” in Japan from the very beginning of medical school. In addition, we also evaluated the educational value of using “Escape Rooms”, a series of timed, game-based scenarios using simulators, as a part of the final assessment of the course.

**Methods:**

At the end of the course, the interview capabilities of 140 first year medical students at International University of Health and Welfare (Japan) were assessed by physicians who acted as simulated patients. Physical examination skills were assessed using the “Escape Room” team task method. Students also self-assessed their confidence in their physical examination skills pre and post “Escape Rooms.” A day prior to the final assessment, students completed an anonymous course evaluation.

**Results:**

The average global rating of the students’ medical interview skills using a rating scale from 1 to 6 (1-fail 6-outstanding, no different from practicing junior physician’s level) was 4.6. Twenty-two students scored the highest mark of 6. An average of 89% of “Escape Room” teams finished all the physical examination tasks correctly within the allotted time. All teams that could not finish in time completed all tasks correctly when given an additional 3 to 5 min. Students’ self-assessed confidence in their physical examination skills increased from 49 to 73 (out of 100) pre and post “Escape Rooms.” In the course evaluation questionnaire, 99% of students answered “this course enhanced their motivation” (response rate 89%) and 99% also answered “this course was interesting and useful” (response rate 86%).

**Conclusions:**

This descriptive study analyzing both quantitative and qualitative data showed that the course not only achieved the intended objectives of successfully conducting comprehensive medical interview and basic physical examination skills, but also enhanced student motivation. “Escape Rooms”, used for the course assessment, in itself enhanced students’ self-perceived physical examination skills and had an added educational value.

## Background

Medical interview and physical examination skills are two of the cornerstones of clinical medicine. Most medical schools have preclinical courses to teach these essential skills to their students. These skills are typically taught independently, or during or following an organ-based curriculum [1]. Medical students appear to be taught these skills at varying times in their education [2]. Role-plays and interviews with simulated patients, followed by feedback and discussion, are one of the widespread and effective teaching methods for teaching interview skills [3]. Overall, there seems to be wide variation in how medical schools teach physical examination to preclinical year students [4, 5].

Historically, teaching medical interview and physical examination has been a challenge for medical schools in Japan [6]. These skills were typically thought of as part of a “hidden curriculum” or “traditional apprenticeship” rather than taught distinctly as part of any official curriculum [6]. In the 2000s, rapid advancement and changes in medical education occurred in Japan, including the introduction of the mandatory two-year structured rotating residency program upon graduation to enhance fundamental clinical skill acquisitions among all graduates [7–9]. In 2005, objective standardized clinical examinations (OSCE) were also introduced as part of the requirement for all medical students in Japan to move from preclinical to clinical years to assure they had sufficient clinical skills before starting their clinical clerkships [7]. Nonetheless, we feel there is still room for improvement in the Japanese preclinical curriculum to further enhance students’ medical interview and physical examination skills.

International University of Health and Welfare (IUHW) School of Medicine opened in April 2017, the first of its kind, 38 years after the Japanese government banned the establishment of new medical schools in Japan. While all other Japanese medical schools teach using only Japanese as an official language, the first 2 years of IUHW School of Medicine’s curricula are taught entirely in English, save for a few courses that are taught bilingually. One in seven students at IUHW are international students, including many full scholarship students from underprivileged Asian countries. From the first day of medical school, freshman entering IUHW School of Medicine take a bilingually (English and Japanese) taught course titled “Medical Interview and Physical Examination I (Basics)”. In addition to the course objectives outlined in the syllabus, which are to be able to conduct a comprehensive medical interview and to conduct a limited physical examination, this early clinical emersion class aims to motivate students as they embark on their journey of becoming physicians; teach rapport building skills through numerous peer practice sessions; and help freshmen begin to visualize and prepare for what clinicians actually do every day in clinics and hospitals.

In Japan, there are very few studies reporting the impacts of introducing medical interview and physical examination from the very beginning of medical school. More specifically, introducing a medical interview and physical examination course as part of the official curriculum, to be taught in both in English and Japanese, is very novel in itself.

Another unique aspect of this course was using “IUHW Escape Room” as part of the end-of-course assessment. IUHW Escape Rooms are a series of timed, game-based clinical scenarios using simulators that assess the students’ basic physical examination skills learned during the course, namely blood pressure taking skills, cardiovascular examination skills, and pulmonary examination skills. Students also learned Basic Life Support (BLS) during their freshman orientation; therefore, we also assessed their BLS skills at the end of this course. Escape rooms, since their inception in Japan in 2007, are a popular type of adventure game in which a team of players discover clues and solve puzzles in order to accomplish a goal within an allotted time. Recently, there has been a rise in introducing “Escape Rooms” in medical education [10–13]. Nonetheless, there is a paucity of literature regarding the use of “Escape Rooms” in the assessment of medical students’ clinical skills. According to the systemic review of 44 gamified learning studies conducted by van Gaalen A.E.J. et al. in 2021, there was no study that focused on physical examination techniques and skills [13].

With this background in mind, this study aimed to describe and explore the achieved competencies and educational impact among the medical students through this newly introduced course.

## Methods

This is a descriptive study to analyze both quantitative and qualitative data which was collected among our medical students who were registered for this course and participated in the final summative assessment of the course. The educational impact of this course among our students was analyzed using the Kirkpatrick’s four level-model [14].

For the medical interview assessment, an assessment check list and global rating were utilized. This assessment check list, global rating system (1–2: fail to 6: outstanding, no different from practicing junior physician’s level) and course evaluation forms have been institutionally developed over the past 4 years for approximately 560 1st year students. Feasibility and practicality were adjusted and improved annually. We found this process was sufficient enough to be considered a pilot study before this study. Interrater reliability was assured by introducing “double” assessment by the two faculty members for the global rating of 1–2 (fail) and 6 (outstanding as a resident level). This means that students who were rated as 1–2 or 6 were assessed by at least two faculty members.

### General information about the course

“Medical Interview and Physical Examination 1 (Basic)” is a 30-h required course for all freshmen at IUHW School of Medicine. The three course objectives are: 1. Observe patient-health professional hospital communication to deepen understanding of medical interview, 2. conduct comprehensive medical interviews while building rapport, and 3. conduct basic general physical examinations.

The 30-h course was divided into 5 sessions: 5 total hours of lecture-based classes on medical interview; 3 total hours of lecture-based classes on physical examination; 10 total hours of practice sessions for both medical interview and physical examination; 6 h of early exposure in the hospital in small groups; and 6 h at the end of the course for medical interview and physical examination summative assessment.

During the 10 total hours of practice sessions, in addition to group and peer practice sessions, all students underwent a one-on-one practice session with a faculty member to improve their medical interview skills. For physical examination skills, students had small group sessions to practice skills with faculty members. In addition, at the final medical interview assessment, after the summative assessment, each student received a formative immediate feedback from their evaluator for 5 to 10 min.

This class is taught bilingually using both Japanese and English. The course director is a Japanese physician trained in the U.S. who practiced and taught medicine as an attending physician at U.S. medical schools for over 15 years. Over the past 4 years, faculty members who have taught this course include not only Japanese physicians, but also international physicians who received their training in England, Egypt, the Philippines, and Bangladesh. In their second year, students take “Medical Interview and Physical Examination 2 (Advanced)” course which builds upon this introductory freshman course with more advanced skills. Students further practice these skills during their clinical clerkships which start from their fourth year at IUHW.

The course grade was calculated using both the final assessments (50%) and class engagement and participation (50%). The final assessments consisted of the observed medical interview score and Escape Room scores for the physical examination skills. Class engagement and participation was assessed by the responses submitted during each class and various course assignments, including a reflection paper on students’ early exposure experiences in the hospital.

### How the course adapted to the COVID-19 pandemic

During the 2020 academic year, this course was offered completely online due to the COVID-19 pandemic. During the 2021 academic year, this course was offered on campus as it was prior to 2020; however, in accordance with IUHW COVID-19 infection control policy, instead of using one classroom to accommodate a class of 140 students, we used two classrooms to reduce the occupancy rate to 50% for social distancing. Faculty enforced a constant mask mandate; careful hand hygiene with alcohol-based disinfectant; and adequate ventilation. All students, faculty, and staff were subject to infrared temperature screening each time they entered the medical school building.

### Assessment logistics of the course for 2021

**Fig. 1:**
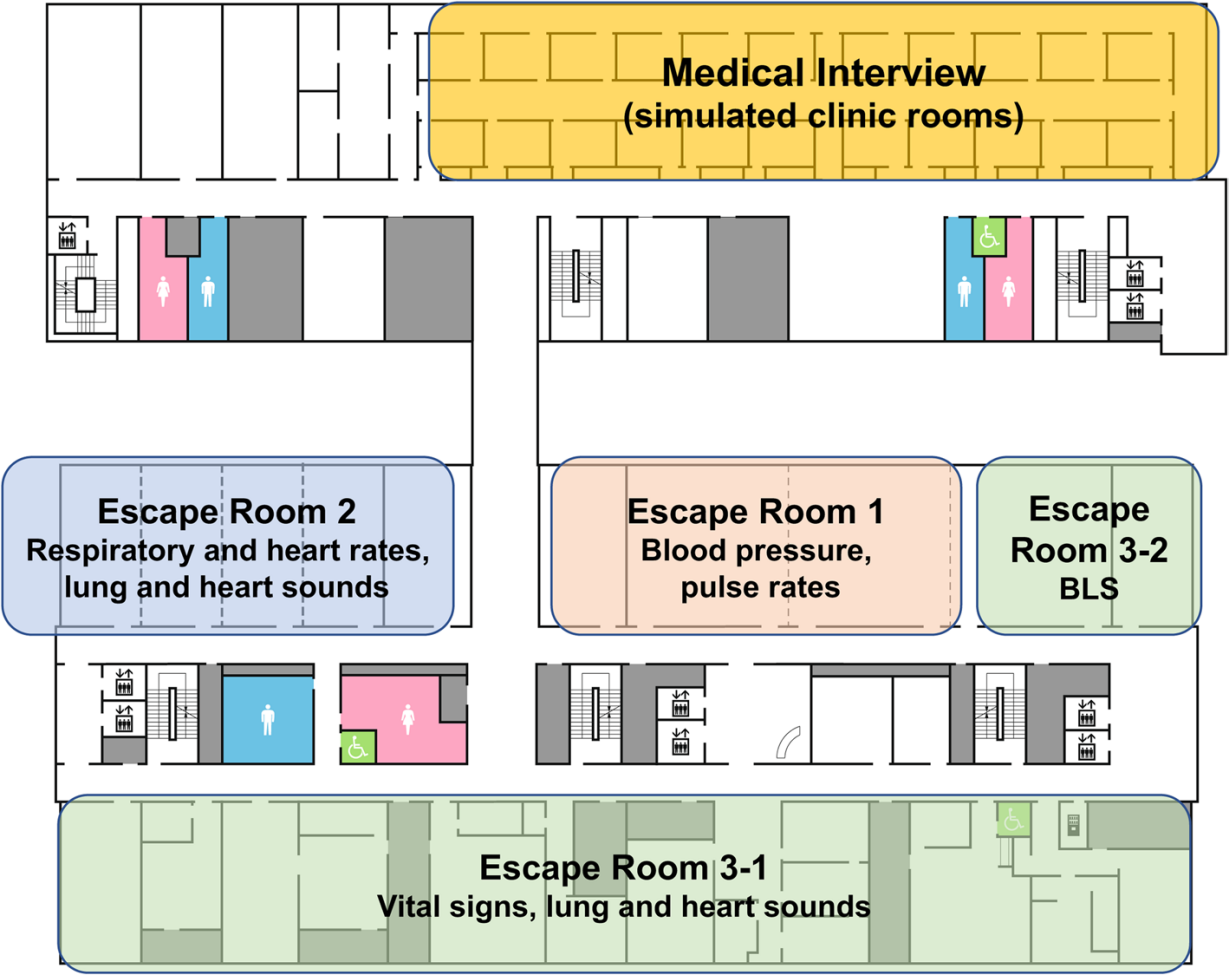
Floor plan of IUHW Simulation Center where the final assessment was conducted. Four stations were used for the final assessment of the course in IUHW Simulation Center. For medical interview assessment, each student performed medical interview with a simulated patient in the simulated clinic room. In Escape Room 1, each student measured blood pressures of classmates and an arm simulator. In Escape Room 2, each student measured and evaluated respiratory rates, heart rates, lung and heart sounds using simulators. Escape Room 3, in the first section, students evaluated four simulators with four different scenarios, and in the second section, they were evaluated for quality CPR

Medical interview skill assessment consisted of 10 to 13-min medical interview using simulated patients (SPs). Before COVID-19, SPs for this assessment were volunteers from the community trained by IUHW School of Medicine staff. Since some of our SPs were not yet fully vaccinated for COVID-19 by the time of the 2021 assessment, we decided not to ask our community SPs to participate in this in-person course assessment. Prior to the pandemic, we also had external evaluators such as practicing clinicians from our affiliated hospitals to have two total evaluators for each student. However, in 2021, instead of four people in the simulated examining room (two evaluators, one SP, and one student), for social distancing, we had the evaluators also serve as SPs, thus reducing the number of people in each simulated examining room to two. SPs were trained for several months by our faculty members on cases used for the examination to ensure consistency. Faculty members functioning in SP roles were given detailed case instructions specifying exactly what to answer for each anticipating student’s interview question. In addition, faculty members were familiar with their roles as SP as they have either played SP roles prior to this study or have observed the students’ practice interview sessions with SPs prior to this study. We video recorded all interviews and had the recordings of the highest-scoring (6 out of 6) and the lowest-scoring (2 out of 6) student performances independently reviewed by another evaluator. Using eight simulated examining rooms at our Simulation Center, 8 evaluators assessed 3 students per hour (Fig. 1). We used different cases for each hour to avoid students sharing their cases with other students. Ten minutes before the start of an hour, the students being assessed for the next hour were kept in a separate room from other students with no cell phone or computer access to avoid any communication with other students being assessed during the same hour.

To evaluate the physical examination skills students learned and practiced during this course, we created several “IUHW Escape Rooms” at our Simulation Center (Fig. 1). Students were divided randomly into groups of three. The grouping was announced on the morning of the assessment. In each Escape Room, students had to complete the assigned tasks correctly with their team members within the allotted time.

In Escape Room 1 (Table 1), each student measured the blood pressure of their two team members, two classmates from other groups, and one simulator arm. Five blood pressure simulators (B.P/Pulse Assessment Simulator (BT-CEAB2), BT Inc., Gyeonggi-do, Korea) were available for maximum 48 students at any time in this Escape Room 1. Each arm had a different set value. The accuracy of blood pressure was assessed using the simulator’s set blood pressure value ±10 mmHg. Students were allowed to repeat the measurement as many times as they wished using the allotted time until all the team members completed the task.

**Table 1:** Three Escape Rooms for conducting basic and limited general physical examinations

Room	Physical examination skills	Subjects
Escape room 1	blood pressures, pulse rates	classmates, blood pressure simulators
Escape room 2	respiratory rates, heart rates, lung and heart sounds	heart sound simulators, lung sound simulators
Escape room 3–1	vital signs, lung and heart sounds	high fidelity human patient simulators with 4 scenarios
Escape room 3–2	basic life support	BLS training mannequins

In Escape Room 2 (Table 1), students evaluated five respiratory simulators which were set up for four cases (asthma, pneumothorax, congestive heart failure, bradypnea) and one normal setting. Similarly, students evaluated five cardiac simulators with four cases (aortic regurgitation, atrial fibrillation, PSVT, and bradycardia) and one normal setting. Each student measured respiratory rate and evaluated the lung sounds for respiratory simulators, heart rate and heart sounds for cardiac simulators. Students were expected to discuss and conclude whether the findings for each simulator were “normal” or “abnormal”. If abnormal, each team was asked to describe what kind of abnormalities they found. Students were not expected to provide any diagnosis or any pathophysiology leading to the abnormalities, but simply describe why they thought the findings were not normal. Ten respiratory simulators (Lung Sound Auscultation Trainer “LSAT” ver.2, Kyoto Kagaku Co. Ltd., Kyoto, Japan) and ten cardiac simulators (Cardiology Patient Simulator “K” version 2, Kyoto Kagaku Co. Ltd., Kyoto, Japan) were used for Escape Room 2 for a maximum of 16 groups at any time.

Escape Room 3 had two sections (Table 1). The first section was to assess four simulators with four scenarios (anaphylactic shock, left pneumothorax, COVD-19 pneumonia, and hypovolemic shock due to the postpartum bleeding). Student read a brief medical history that was posted next to each simulator and assessed the vital signs, lung and heart sounds. The second section was set up to perform a high-quality chest compression for 5 min as a team. Of note, freshmen have learned BLS (Basic Life Support) as part of their Freshmen Orientation. The quality of chest compression was evaluated by QCPR (Resusci Anne QCPR, Laerdal Medical, Stavanger, Norway). The set value was 80 points or more to pass this task. For a maximum of 24 groups at a time, 12 QCPR mannequins (Resusci Anne QCPR, Laerdal Medical, Stavanger, Norway) and 12 simulators (SimMan® 3G, Laerdal Medical, Stavanger, Norway, Resusci Anne Simulator, Laerdal Medical, Stavanger, Norway, Physical Assessment Simulator Physiko, Kyoto Kagaku Co. Ltd., Kyoto, Japan, and NOELLE® S2200 VICTORIA™, Gaumard Scientific, Miami, Florida, U.S.) were prepared for Escape Room 3.

Students were asked to complete each Escape Room task within 1 h. Each Escape Room had faculty members for technical support and scoring after each team had completed the task. For each Escape Room, each team was allowed to repeat the tasks, within the allotted hour, as many times as they needed until they passed the requirement in order to move to the next Escape Room. For infection control, students and faculties followed 100% mask use, adhered to strict hand hygiene rules, kept the windows open, and wiped the mannequins down every hour. Disposable gloves were also available. Students had a debriefing session after all Escape Rooms were completed. All cases were reviewed and the correct physical assessments were discussed. Underlying diagnosis and pathophysiology for each case was also explained at a level appropriate for the 1st trimester 1st year medical student with no prior medical knowledge.

In addition to the evaluation of the content of the course itself, we also evaluated whether the use of “Escape Rooms” during the end-of-course assessment improved student competency in itself. To do so, we looked at not only the evaluators’ assessment of student performance, but also the students’ perceived self-confidence in their physical diagnosis skills before and after the “IUHW Escape Rooms”.

### How students were assessed whether they met the course learning objectives

#### Course learning objective 1. Students are able to conduct comprehensive medical interviews

A total of 10 components of comprehensive medical interviews (self-introduction, checking the patient’s full name and date of birth, obtaining consent for an interview, asking history of present illness, asking past medical history, asking medication history, asking allergies, asking family history, asking social history, summarizing the history to patient and asking for any questions) were independently marked as “done” or “not done” by the evaluator.

#### Course learning objective 2. Students are able to conduct basic and limited general physical examinations (vital signs, cardiac exams and pulmonary exams) covered during this course and high-quality chest compression learned during the freshman orientation

Each group of 2–3 students received a group grade for each “Escape Room” task based on the accuracy of the performed task and the time it took to complete the task.

### How students were assessed for their rapport building skills at the end of this course

In the final assessment of the course, students interviewed SPs, and the SP-acting evaluator graded each student’s rapport building skills using a scale from 0 (Fail) to 6 (Outstanding).

### How the educational value was evaluated for “escape rooms”

Students completed the pre- and post- self-evaluation regarding their confidence in their ability to perform the physical examination skills learned during the course and assessed using Escape Rooms.

### How the course was evaluated whether it achieved the intended additional goals

#### Students felt this course enhanced their motivation

Students completed an anonymous “Medical School Course Evaluation Questionnaire” during the final class, prior to the final assessment, using a rating scale from 1 to 6.

#### Students felt this course was interesting and useful for their future career

Students completed an anonymous “University-wide Course Evaluation Questionnaire” during the final class, prior to the final assessment, using a rating scale from 1 to 4.

## Results

Summary of results is shown in Table 2.

**Table 2:** Summary of results

Average course grade out of 100 (range)		90.9 (60.3–100)
Course passing rate		100%
Average global rating for medical interview score of 1 to 6 (1-Fail, 2-Marginal fail, 3- Marginal pass, 4-Pass, 5-Above expected standard, 6-Outstanding, no different from practicing junior physician’s level)		4.6
Students who were able to conduct comprehensive medical interview		97%
Number of teams completed Escape Room tasks within allotted time (47 total teams participated)	Escape Room 1	42
	Escape Room 2	40
	Escape Room 3	45
Self-assessed students’ average confidence in their physical examination skills before the Escape Room (0-not at all confident, 100-very confident) (IQR)		49 (35–60)
Self-assessed students’ average confidence in their physical examination skills after the Escape Room (0-not at all confident, 100-very confident) (IQR)		73 (60–81)
Students who reported that the course enhanced their motivation		99%
Students who thought that the course was useful for their future		99%

### Course attendance rates and final course grades

There were 140 freshmen registered for this course. Average attendance rate during the course was 95%, as determined by the short answers or reflections students were asked to submit during each class. The average course grade was 90.9 (range 60.3–100) and all students passed. One hundred thirty-four students underwent the final assessment as scheduled in July. Six students took the retake assessment due to excused absences.

Knowledge, skills, and attitudes learned during the course were assessed using the Miller’s pyramid at the level of “Shows how” [15]. The average global rating for the student’s medical interview using the score of 1 to 6 was 4.6. (1-Fail, 2-Marginal fail, 3- Marginal pass, 4-Pass, 5-Above expected standard, 6-Outstanding, no different from practicing junior physician’s level). Four students scored 2 (borderline failure) (3%), and 22 students scored 6 (at a practicing junior physician’s level) (16%). Video recordings of 3 students with borderline failure and 19 students with an outstanding evaluation of 6 out of 6 were independently reviewed by the course director, and the ratings were confirmed to be accurate. The video recording of one student with borderline failure and 3 students with an outstanding evaluation were reviewed by another evaluator since these 4 students were originally evaluated by the course director. The ratings for these were also confirmed to be accurate.

### Assessment of the course learning objective 1. Students are able to conduct comprehensive medical interviews

All students were able to accomplish all these tasks 100% of the time (“done”), except for 3 students forgetting to check the patient’s full name and date of birth and one student forgetting to ask the social history. For review of systems, since the medical interview assessment was limited to 10 to 13 min, students were instructed in advance that they were allowed to perform an abbreviated version of the review of systems: as long as they ask more than 3 different system-based questions in addition to the questions related to the chief complaint, that will be sufficient to be marked as students did ask review of system questions. Since this was an abbreviated review of systems, we were not able to fully assess the comprehensiveness of students’ ability to conduct review of systems during this assessment.

### Assessment of the course learning objective 2. Students are able to conduct basic and limited general physical examinations (vital signs, cardiac exams and pulmonary exams) covered during this course and high-quality chest compression learned during the freshman orientation

Out of 47 teams, 42 teams for Escape Room 1, 40 teams for Escape Room 2, and 45 teams for Escape Room 3 finished all tasks within the allotted time with passing scores. Those teams that could not finish all tasks within the allotted time did complete them with an additional 2 to 5 min. The mean time taken to pass all required tasks was 50 min (IQR 44–54) for Escape Room 1 and 56 min (IQR 52–60) for Escape Room 2. For Escape Room 3, scoring time influenced the “escape” (passing completion) time, so we could not accurately determine the exact time taken by each team.

### Assessment of the students’ rapport building skills

Evaluators were asked to grade the students’ rapport building using the scale of 1 (no rapport) to 6 (outstanding rapport building). However, since the assessment of the rapport building itself was not used for the final course grade, and was incorporated into the final global medical interview assessment, some evaluators forgot to grade this item. As a result, only 120 out of 140 students (86%) had rapport item marked. For these 120 students, the average rapport building score was 4.6. Nineteen students (16%) received 6, 43 students (36%) 5, 45 students (38%) 4, 12 students (10%) 3, 1 student (1%) 2, and 0 students (0%) 1.

### Evaluation of the educational impact of “escape rooms”

Students completed the pre- and post-Escape Rooms regarding their self-assessed confidence in their physical examination skills taught during this course and evaluated by these Escape Rooms. Pre-Escape Room average score was 49 (0-not at all confident to 100-very confident) for 132 students completed this pre-survey. (IQR 35–60). Post-Escape Room average score obtained during the debriefing session improved to 73 (IQR 60–81) for 109 students completed the post experience survey.

### Evaluation of the intended additional goal 1. Students will feel this course enhanced their motivation

A total of 124 students completed the end of course anonymous “Medical School Course Evaluation” prior to the final assessment using the standard medical school questionnaire format. For the question “Did you find this course motivating?” students were instructed to choose one answer from six options (I think so very much, I think so, I think so little, I do not think so very much, I do not think so, I do not think so at all). Fifty-two students (42%) answered “I think so very much”, 51 students (41%) answered “I think so”, 20 students (16%) answered “I think so a little”, indicating that 99% of the students were motivated by this course.

### Evaluation of the intended additional goal 2. Students will feel this course was interesting and useful for their future

A total of 121 students (86%) completed the end of the course anonymous “University Course Evaluation” using the standard University questionnaire format prior to the assessment. For the question “This course was interesting and it made me feel it will be useful for my future” students were instructed to choose one answer from 4 choices (I think so, I think so a little, I do not think so very much, I do not think so). One hundred eight students (89%) answered “I think so” and 12 students (10%) answered “I think so a little”, indicating that 99% of the students felt this course was interesting and useful.

## Discussion

Summative assessments were used for medical interview and physical examination skills using the Miller’s pyramid at the level of “Shows how” [15]. The educational impact of this course among our medical students was investigated using the Kirkpatrick’s four level-model [14]. In this study, we show that teaching medical interview and physical examination from the very beginning of medical school achieved intended both the course objectives (students will be able to conduct comprehensive medical interviews and be able to conduct basic and limited general physical examinations: vital signs, cardiac exams and pulmonary exams) and additional goals (students felt that this course enhanced their motivation, was interesting and was useful for their future). Of note, this end of course evaluation is used University-wide to award the “Best Teaching Award” each year. This course was awarded the campus-wide “Best Teaching Award” last year, as it received very positive feedbacks from students on last year’s survey. (This year’s award will not be announced until the end of this academic year.)

Historically, incorporating a formal, dedicated course for medical interview and physical examination skills in Japanese medical schools has been a challenge [16]. As part of the major reform in undergraduate and postgraduate medical education, the OSCE was formally adopted in Japan in 2005 [7]. As a result, many medical schools in Japan began formally teaching students medical interview and physical examination skills, usually during the last pre-clinical year, just before students need to take the OSCE. However, there are only a few medical schools that teach the importance of medical interview and physical examination in an integrated manner from early preclinical years. From this point of view, this course is unique especially in Japan as we offer extensive, dedicated, even bilingual courses on medical interview and physical examinations skills to our first- and second-year students.

Different opinions exist regarding the value of teaching clinical skills such as medical interviewing and basic physical diagnosis skills early on without any medical knowledge.

Those who discourage such an early education may argue that there is little value if students merely learn the mechanics or techniques without understanding what they are doing. We found that it is possible for the students to learn these skills even without medical knowledge as previously reported [17]. Obviously, students must continue to learn and master both the skills and reasoning necessary to perform medical interview and physical examination throughout their medical education.

Due to the COVID-19 pandemic, having medical students in clinics and hospitals is becoming ever more challenging. It is imperative that clinical year students complete their required clinical clerkships despite the pandemic to graduate with adequate clinical training. However, clinical exposure for pre-clinical year students is often deprioritized for the sake of public safety and health in clinics and hospitals. Although there is no substitute for the real patient encounter, simulation-based medical education, for instance, has been extensively studied and reported to be effective for learning among medical students [18]. Introducing medical interview and physical examination course very early during the freshman year like what we offered in this course, could potentially also serve as an alternative to the real “early exposure” clinical rotation for freshman when clinical capacities are limited. On the basis of our study, early in-class clinical simulation course seems to have similar motivating effects by exposing students to the cornerstones of clinical medicine: talking to and examining patients.

Medicine cannot be practiced in vacuum, and communication is a skill as important as solid medical knowledge for practicing physicians. Some students enter medical school with previously developed outstanding communication skills, while others need vast improvement. Although we did not measure the improvement of each student’s communication skills before and after this course, on average, the rapport building skills assessed by the evaluators at the end of the course was 4.6 using the 0 to 6-point scale. It is worth mentioning that the majority of the students did not know the word “rapport” when they started this course. Like any skills, good communication takes practice. From the start of their medical school experience, students learned through this course about the meaning of rapport and how to build rapport with others. Since it is beyond the scope of this paper, future study is needed to assess whether learning and practicing rapport building skills through medical interviewing course will also help students’ communication skills with their peers, which is particularly important as they build their learning community early in their medical school life.

Assessment has been strategically designed and utilized to promote deeper learning among students [19]. To improve and change the quality of learning among students, shifting from “Assessment of learning” [20] to “Assessment for learning” [19] has been advocated since approximately two decades ago. “Escape Rooms” are an engaging tool for active learning and are being used more and more in medical education as of late. By using “Escape Rooms” as part of the final assessment of the course, we demonstrated that the students felt their skills and confidence improved based on the pre- and post- Escape Room assessment. This improvement indicated Kirkpatrick’s educational impact level-1(reflection) [14]. Further studies are needed to explore the deeper or higher-level educational impact over a longer-time period such as the time when the students start their clinical clerkships.

### Limitations of the study

This study was descriptive in nature to demonstrate our students’ objectively assessed competencies by the faculty and subjective perceptions among the students using the online questionnaire. Further investigations for generalizability and transferability are needed to explore the key factors and conditions for successfully implementing medical interview and physical examination course at the very beginning of medical school and the summative assessment using the Escape Rooms for the course.

## Conclusions

Medical interview and physical examination are essential skills for every physician. While there is no consensus as to the best timing to teach these skills to medical students, this study demonstrated that it is possible to teach these skills and have students achieve intended competencies very early in their medical education. It also has additional benefits including motivating students. As assessment can be strategically designed to enhance learning, incorporating “Escape Rooms” to the final assessment also proved to have an educational value in itself.

## Data Availability

The datasets generated and analyzed during the current study are not publicly available since they are students’ assessment data, but are available from the corresponding author on reasonable request.

## Abbreviations

IUHW: International University of Health and Welfare
SP: Simulated Patient
BLS: Basic Life Support
OSCE: Objective Standardized Clinical Examinations
